# P-660. Viral Pathogens among Hospitalized Adults enrolled in the Acute Respiratory Illness in Adults (ARIA) Study : Pilot phase results from a prospective cohort study

**DOI:** 10.1093/ofid/ofaf695.873

**Published:** 2026-01-11

**Authors:** Jennifer L Kuntz, Mark A Schmidt, Holly C Groom, Jennifer K Meece, Richard A Mularski, John F Dickerson, Karen Jacobson, Amy Wiesner, Courtney Oxandale, Weiming Hu, Maureen O’Keeffe-Rosetti, Lisa Glasser, Sudhir Venkatesan, Carla Talarico, Nicola Klein

**Affiliations:** Kaiser Permanente Center for Health Research, Portland, Oregon; Center for Health Research, Kaiser Permanente Northwest, Portland, Oregon; Kaiser Permanente Center for Health Research, Portland, Oregon; Marshfield Clinic Research Institute, Marshfield, Wisconsin; 1. Kaiser Permanente Center for Health Research, Portland, Oregon, Portland, Oregon; Kaiser Permanente Center for Health Research, Portland, Oregon; Kaiser Permanente Vaccine Study Center, Division of Research, Oakland, California; Kaiser Permanente Vaccine Study Center, Division of Research, Oakland, California; Kaiser Permanente Vaccine Study Center, Division of Research, Oakland, California; Kaiser Center for Health Research, Portlannd, Oregon; Kaiser Permanente Center for Health Research, Portland, Oregon; AstraZeneca, Wilmington, DE; Medical and Payer Evidence Statistics, BioPharmaceutical Medical, AstraZeneca, Cambridge, UK, Cambridge, England, United Kingdom; Vaccines and Immune Therapies, BioPharmaceuticals Medical, AstraZeneca, Gaithersburg, MD, USA, Gaithersburg, Maryland; Kaiser Permanente Vaccine Study Center, Division of Research, Oakland, California

## Abstract

**Background:**

Acute respiratory illness (ARI) causes significant morbidity and mortality worldwide, although gaps remain in understanding the burden of ARI due to respiratory syncytial virus (RSV) and human metapneumovirus (hMPV). The Acute Respiratory Infections in Adults (ARIA) is a two-year prospective study, with a pilot phase intended to determine the feasibility of assessing the burden of acute respiratory illness (ARI) in adults. Here we report pilot phase data between November 18, 2024 and March 31, 2025.

**Figure:**
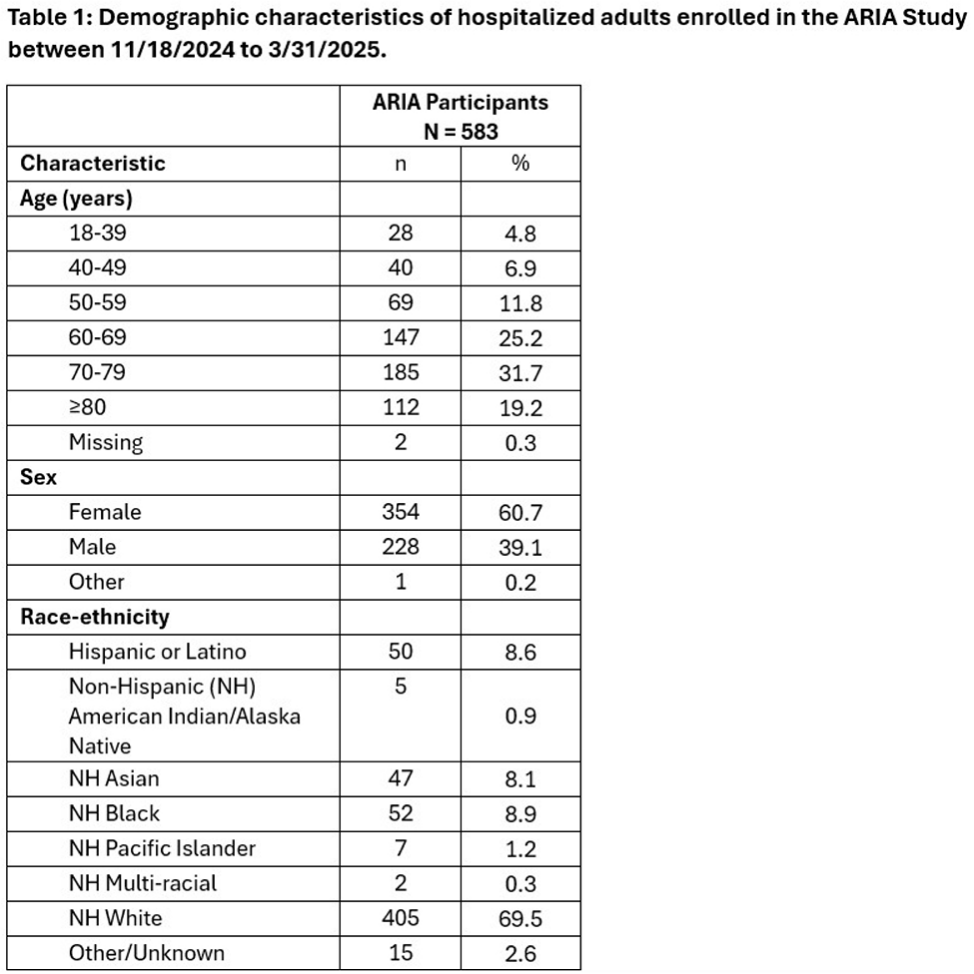

**Figure:**
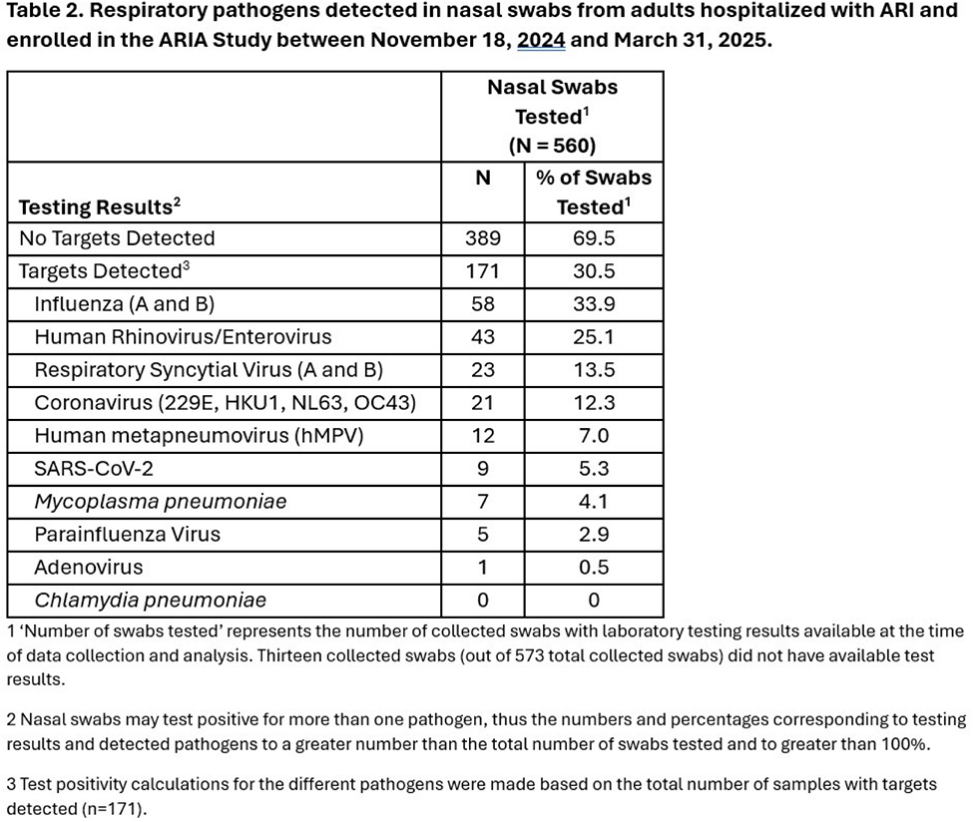

**Methods:**

We recruited Kaiser Permanente Northwest and Northern California members ≥18 years hospitalized due to ARI from 11/18/2024 to 3/31/2025. Nasal swabs from enrollees underwent real-time PCR testing for respiratory pathogens using the GenMark ePlex RP2 multiplex panel at Marshfield Clinic Research Institute. Here we describe population characteristics and respiratory pathogen test results.

**Results:**

Of 2,043 eligible members, we enrolled 583 (29%) and collected nasal swabs from 573 (98%). Most participants were female and were aged 60-79 years (Table 1). Of 171 (30.5%) swabs with pathogens detected, the most common were: influenza (A or B, 33.9%), human rhinovirus/enterovirus (25.1%), RSV (A or B, 13.5%), coronaviruses (12.3%), and hMPV (7.0%) (Table 2). SARS-CoV-2 was detected in 5.3% of specimens. During this partial season, influenza positivity rate among hospitalized participants peaked over the two weeks from 02/02/25 to 02/15/25; RSV peaked from 1/12/25 to 1/18/25; and hMPV positivity rates started to increase towards the end of our observation window ( 3/23/25 to 3/29/25).

**Conclusion:**

In this pilot phase analysis from the 2024-2025 respiratory season, influenza was the leading respiratory infection. This pilot phase analysis demonstrates the feasibility of assessing the ARI burden in hospitalized adults, with observed positivity rates similar to community rates and influenza positivity rates peaking during multiple periods, RSV peaking earlier than hMPV. The ARIA study will provide valuable insight into the burden and severity of ARI due to RSV and hMPV among hospitalized adults over a two-year study period.

**Disclosures:**

Jennifer L. Kuntz, MS, PhD, Astra Zeneca: Grant/Research Support|Moderna, Inc.: Grant/Research Support|Pfizer: Grant/Research Support Mark A. Schmidt, PhD, MPH, AstraZeneca: Grant/Research Support|HilleVax: Grant/Research Support|Janssen: Grant/Research Support|Moderna: Grant/Research Support Holly C. Groom, MPH, AstraZeneca: Grant/Research Support|Moderna: Grant/Research Support Jennifer K. Meece, PhD, CSL Seqirus: Grant/Research Support|GSK: Grant/Research Support|ModernaTX: Grant/Research Support Richard A. Mularski, MD, MSHS, MCR, AstraZenica: Grant/Research Support|Pfizer, Inc: Grant/Research Support|Sanofi: Grant/Research Support John F. Dickerson, PhD, AstraZeneca: Grant/Research Support|HilleVax: Grant/Research Support|Moderna: Grant/Research Support Karen Jacobson, MD, MPH, AstraZeneca: Grant/Research Support Weiming Hu, MS, AstraZeneca: Grant/Research Support|Janssen: Grant/Research Support Maureen O'Keeffe-Rosetti, MS, Astra Zeneca: Grant/Research Support|HilleVax: Grant/Research Support|Moderna: Grant/Research Support|Pfizer: Grant/Research Support Lisa Glasser, MD, AstraZeneca: Stocks/Bonds (Public Company) Sudhir Venkatesan, MPH, PhD, AstraZeneca: Stocks/Bonds (Public Company) Carla Talarico, PhD, MPH, AstraZeneca: Stocks/Bonds (Private Company) Nicola Klein, MD, PhD, AstraZeneca: Grant/Research Support

